# High 1-year risk of stroke in patients with hepatocellular carcinoma: a nationwide registry-based cohort study

**DOI:** 10.1038/s41598-021-89867-0

**Published:** 2021-05-17

**Authors:** Jin-Yi Hsu, Peter Pin-Sung Liu, An-Bang Liu, Huei-Kai Huang, Ching-Hui Loh

**Affiliations:** 1 Center for Aging and Health, Hualien Tzu Chi Hospital, Buddhist Tzu Chi Medical Foundation, Hualien, Taiwan No. 707, Sec. 3, Chung Yang Rd., 97002; 2 School of Medicine, Tzu Chi University, Hualien, Taiwan; 3 Department of Neurology, Hualien Tzu Chi Hospital, Buddhist Tzu Chi Medical Foundation, Hualien, Taiwan; 4 Department of Family Medicine, Hualien Tzu Chi Hospital, Buddhist Tzu Chi Medical Foundation, Hualien, No. 707, Sec. 3, Chung Yang Rd., 97002 Taiwan; 5 Department of Medical Research, Hualien Tzu Chi Hospital, Buddhist Tzu Chi Medical Foundation, Hualien, Taiwan

**Keywords:** Cancer, Risk factors, Stroke, Liver cancer

## Abstract

Patients with hepatocellular carcinoma (HCC) might be more vulnerable to develop stroke than other cancer patients because of HCC-associated coagulation dysfunction. However, limited studies have investigated the relationship between HCC and stroke. This nationwide population-based cohort study enrolled all patients with HCC diagnosed between 2011 and 2015 from the Taiwan Cancer Registry and Taiwan National Health Insurance Research Database; an age- and sex-matched cohort without cancer was included. The primary outcome was the 1-year risk for first-ever stroke after the index date. The Fine and Gray competing risk regression model was used to estimate the 1-year stroke risk with adjusted hazard ratios (aHRs). After propensity score matching, each cohort has 18,506 patients with similar baseline characteristics. Compared with the cancer-free cohort, the aHRs in the HCC cohort for overall, ischemic, and hemorrhagic strokes were 1.59 [95% confidence interval (CI), 1.35–1.88], 1.38 [95% CI, 1.15–1.65], and 2.62 [95% CI, 1.79–3.84], respectively. On subgroup analysis, HCC patients without cirrhosis, those with stage 3 or 4 cancer had a higher stroke risk than cancer-free cohort. Therefore, stroke prevention should be considered in patients with HCC, especially in those without cirrhosis and with stage 3 or 4 cancer.

## Introduction

Hepatocellular carcinoma (HCC) is one of the most common type of cancer worldwide^1^. Although HCC is historically lethal, the 5-year survival rate has increased to nearly 50% because of better surveillance strategies and surgical management of HCC^2^. Previous studies have suggested that cancer is a potential risk factor for stroke; the risk of stroke varies depending on the cancer type, cancer stage, and duration after the cancer diagnosis^3–5^. The main mechanism for cancer-related stroke is cancer-associated hypercoagulability resulting from an abundance of hypercoagulable materials, tissue factors, or mucins secreted by cancer cells^6,7^. Moreover, chronic disseminated coagulopathy promotes bleeding tendency because of the extended consumption of coagulation factors^8^. In addition to cancer-associated coagulopathy, patients with HCC often have coagulation dysfunction because of cirrhosis or accumulation of malignant tumor cells in the liver^9,10^. With regards to the relationship between stroke and cancer, HCC should be considered as an important risk factor for ischemic stroke or hemorrhagic stroke due to coagulation dysfunction. Some studies revealed that HCC cells might be associated with systemic thrombotic and hemorrhagic processes^10,11^. However, to date, there is little evidence to clarify the association between HCC and stroke, both ischemic and hemorrhagic. Only one previous observational study analyzed the association between HCC and stroke in their subgroup analyses. However, this study was not specifically designed to evaluate the association between the risk of stroke and HCC, and thus did not adjust for important confounders related to the risk of stroke in patients with HCC and did not obtain data on cancer stages^12^.

We conducted a nationwide, whole-population, registry-based cohort study to clarify the relationship between HCC and risk of stroke. We hypothesize that patients with HCC might have a higher 1-year risk of stroke compared with cancer-free individuals.

## Results

### Patient characteristics

This study included 33,468 patients with HCC and 66,936 patients with age- and sex-matching as cancer-free individuals. Compared with cancer-free individuals, patients with HCC had a higher Charlson comorbidity index, a higher prevalence of diabetes mellitus, cirrhosis, and a history of major gastrointestinal bleeding (Table S1). After propensity score matching, each cohort had 18,506 patients and the characteristics in each cohort were similar with all standardized difference values < 0.2 (Table 1).

**Table 1:** Baseline characteristics in patients with hepatocellular carcinoma and the cancer-free cohort after propensity score matching.

Characteristics	HCC cohort	Cancer-free cohort	Standardized
	*N* = 18,506	*N* = 18,506	Difference
Age^a^	63.2 (12.1)	62.8 (11.8)	0.029
Male	12,813 (69.2)	13,314 (71.9)	0.059
**Income (NTD)**			
Dependence	3780 (20.4)	3554 (19.2)	0.031
15,800–29,999	8725 (47.2)	9243 (50.0)	0.056
30,000–44,999	3511 (19.0)	3464 (18.7)	0.006
45,000 and above	2490 (13.5)	2245 (12.1)	0.040
Charlson comorbidity index^a^	2.8 (2.7)	2.5 (2.2)	0.127
**Comorbidities**			
Hypertension	8667 (46.8)	8570 (46.3)	0.010
Diabetes mellitus	5257 (28.4)	5181 (28.0)	0.009
Dyslipidemia	5233 (28.3)	4759 (25.7)	0.058
Atrial fibrillation	401 (2.2)	302 (1.6)	0.040
Valvular heart disease	135 (0.7)	113 (0.6)	0.015
Congestive heart failure	758 (4.1)	624 (3.4)	0.039
Coronary artery disease	2745 (14.8)	2462 (13.3)	0.044
Peripheral arterial occlusion disease	384 (2.1)	278 (1.5)	0.044
Cirrhosis	5946 (32.1)	5935 (32.1)	0.001
Chronic kidney disease	975 (5.3)	904 (4.9)	0.018
Major gastrointestinal bleeding	175 (1.0)	216 (1.2)	0.022
**Medication use**^**b**^			
Antithrombotic therapy^c^	2293 (12.4)	1937 (10.5)	0.060
Anticoagulant	231 (1.3)	176 (1.0)	0.029
Antiplatelet	2101 (11.4)	1790 (9.7)	0.055

Data are expressed as n (%) unless otherwise indicated.

*NTD* New Taiwan dollar, *HCC* hepatocellular carcinoma, *SD* standard deviation.

^a^Expressed as the mean (SD).

^b^Medication use means a drug prescription for more than 30 days during the observation period.

^c^Antithrombotic therapy means either anticoagulant or antiplatelet use.

### Risk of stroke

**Figure 1:**
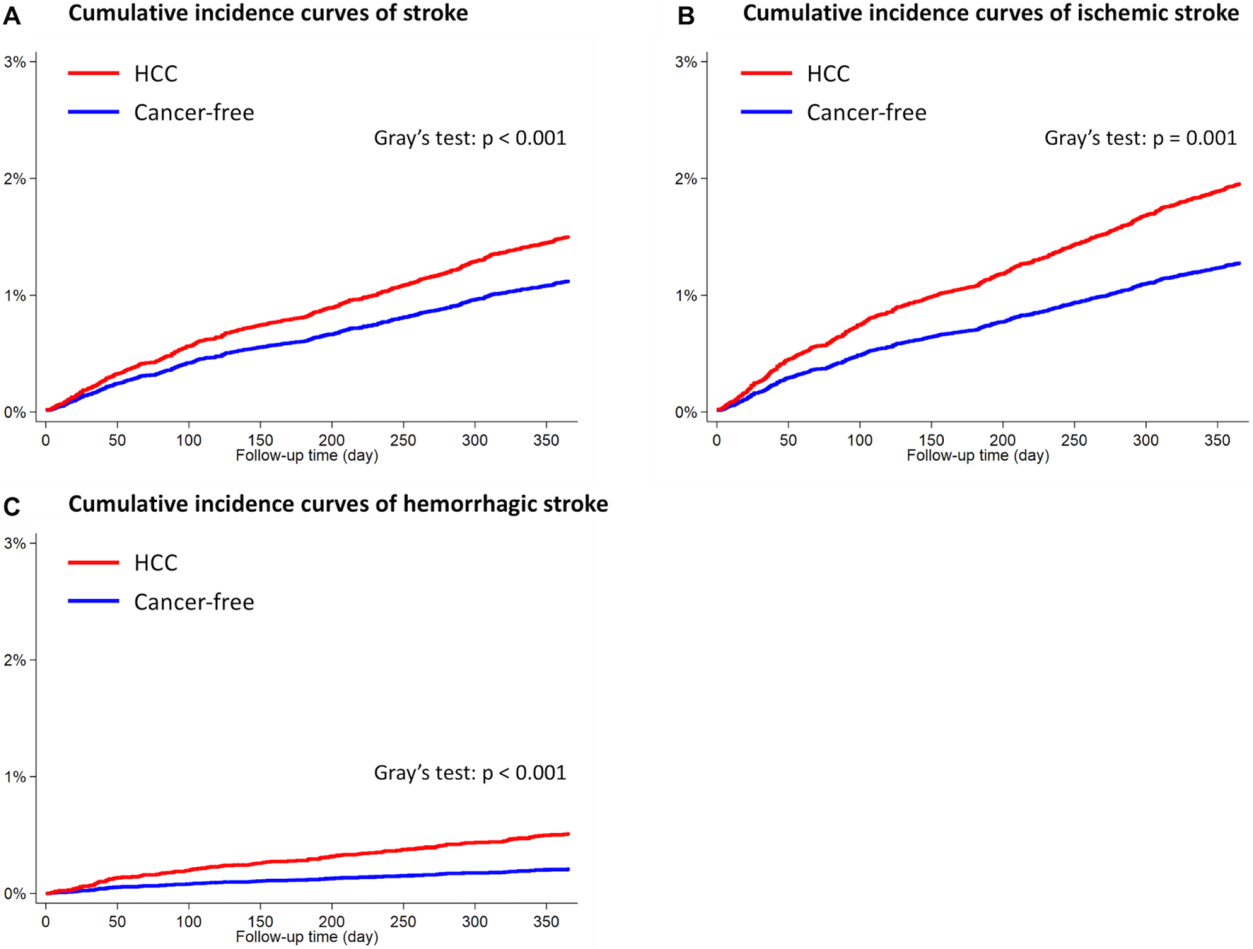
Cumulative incidence curves of stroke in patients with HCC and cancer-free individuals. **(A)** Stroke overall; **(B)** ischemic stroke; **(C)** hemorrhagic stroke. *HCC* hepatocellular carcinoma.

The HCC cohort had 361 patients diagnosed with stroke and the cancer-free cohort had 236 patients diagnosed with stroke 1 year after the index date. The HCC cohort had a higher cumulative incidence of developing stroke overall (Gray’s test, p < 0.001; Fig. 1A), as well as ischemic (Gray’s test, p = 0.001; Fig. 1B) and hemorrhagic (Gray’s test, p < 0.001; Fig. 1C) stroke subtypes, than the cancer-free cohort. The multivariable regression models revealed that HCC was associated with a higher risk of developing stroke (adjusted hazard ratio [aHR], 1.59; 95% CI, 1.35–1.88). The sub-analyses further revealed that the risks of both ischemic stroke (aHR, 1.38; 95% CI, 1.15–1.65) and hemorrhagic stroke (aHR, 2.62; 95% CI, 1.79–3.84) (Table 2) were higher in the HCC cohort compared with those in the cancer-free cohort.

**Table 2:** Risk of stroke in patients with hepatocellular carcinoma compared with the cancer-free cohort after propensity score matching.

Outcome	HCC cohort		Cancer-free cohort		aHR^b^ (95% CI)	*p *value
	Event	IR^a^	Event	IR^a^		
All stroke	361	5.68	236	3.52	1.59 (1.35–1.88)	< 0.001
Ischemic stroke	277	4.36	207	3.09	1.38 (1.15–1.65)	0.001
Hemorrhagic stroke	94	1.48	38	0.57	2.62 (1.79–3.84)	< 0.001

^a^IR per 1000 person-years.

^b^The hazard ratios were calculated using a multivariable Cox proportional hazards regression model with adjustments for income, CCI, comorbidities, and medication use listed in Table 1.

*aHR* adjusted hazard ratio, *CI* confidence interval, *HCC* hepatocellular carcinoma, *IR* incidence rate, *CCI*: Charlson comorbidity index.

### Subgroup analyses for cirrhosis, cancer stages, and cancer treatment

We analyzed the association between HCC and risk of stroke after sub-grouping the HCC cohort according to the presence of cirrhosis, cancer stage, and cancer treatment. The HCC patients in each subgroup were compared with their matched cancer-free individuals. Compared with the cancer-free cohort, HCC patients without cirrhosis, and those with stage 3, stage 4, or an unknown stage cancer had a higher risk of stroke (Table 3). HCC patients also had higher risk of stroke than the cancer-free individuals regardless of receiving cancer treatment. The subgroups analysis for both ischemic stroke (Table S2) and hemorrhagic stroke (Table S3) disclosed similar findings.

**Table 3:** Subgroup analyses to assess risk of stroke in patients with hepatocellular carcinoma compared with the cancer-free individuals according to the presence of cirrhosis, cancer stages, and cancer treatment.

	aHR^a^	95% CI	p value
**Cirrhosis**			
Yes	1.24	0.87–1.75	0.229
No	1.73	1.42–2.09	< 0.001
**Cancer stage**			
1	0.93	0.69–1.26	0.650
2	0.97	0.68–1.39	0.878
3	2.22	1.51–3.27	< 0.001
4	4.90	3.00–7.99	< 0.001
Unknown	2.07	1.33–3.22	0.002
**Cancer treatment**^**b**^			
Yes	1.49	1.23–1.80	< 0.001
No	1.98	1.38–2.84	< 0.001

*aHR* adjusted hazard ratio, *CI* confidence interval, *CCI* Charlson comorbidity index.

^a^The hazard ratios were calculated using a multivariable Cox proportional hazards regression model with adjustments for income, CCI, comorbidities, and medication use listed in Table 1. Cancer-free controls comprised the reference group for analysis, and the information for the cancer-free reference group is not provided in the table.

^b^Cancer treatment means patients had either surgical therapy, chemotherapy, or radiation therapy.

### Sensitivity analyses

The sensitivity analysis, involving all study participants without matching, revealed similar findings as the results after propensity score matching. The HCC cohort still had a higher risk of stroke overall (aHR, 1.68; 95% CI, 1.46–1.94) as well as a higher risk of ischemic stroke (aHR, 1.45; 95% CI, 1.23–1.71) and hemorrhagic stroke (aHR, 2.73; 95% CI, 2.03–3.66) compared with non-cancer cohort (Table 4).

**Table 4:** Risk of stroke in patients with hepatocellular carcinoma compared with the cancer-free cohort without propensity score matching.

Outcome	HCC cohort		Cancer-free cohort		aHR^b^ (95% CI)	*p *value
	Event	IR^a^	Event	IR^a^		
All stroke	603	5.21	664	2.73	1.68 (1.46–1.94)	< 0.001
Ischemic stroke	445	3.85	577	2.38	1.45 (1.23–1.71)	< 0.001
Hemorrhagic stroke	174	1.50	114	0.47	2.73 (2.03–3.66)	< 0.001

*aHR* adjusted hazard ratio, *CI* confidence interval, *CCI* Charlson comorbidity index, *HCC* hepatocellular carcinoma, *IR* incidence rate.

^a^IR per 1000 person-years.

^b^The hazard ratios were calculated using a multivariable Cox proportional hazards regression model with adjustments for income, CCI, comorbidities, and medication use listed in Table 1.

## Discussion

This study demonstrates that patients with HCC have a higher risk of stroke compared with cancer-free individuals. Patients with HCC have a 59% higher risk of all stroke types, with a 38% higher risk of ischemic stroke and a 162% higher risk of hemorrhagic stroke compared with cancer-free individuals. The stroke risk varied based on presence of cirrhosis and different cancer stages; patients without cirrhosis and those with stage 3 or stage 4 cancer had a higher risk of stroke compared with cancer-free individuals. To our knowledge, this is the largest cohort study to specifically investigate the association between HCC and stroke, and the first cohort shows that HCC is associated with higher risk of ischemic stroke.

The association between cancer and stroke has been reported in many common cancers, such as lung cancer^4,12,20^, breast cancer^4,12,20^, colorectal cancer^4,12,20^, prostate cancer^4,12,20^, and stomach cancer^4,12^. HCC is one of the common types of cancer worldwide, but few reports discuss the relationship between HCC and stroke^12^. HCC has high mortality rate; this poor statistic has prevented research concerning the association between HCC and stroke. In Taiwan, the 5-year survival rate of HCC is higher than 50%^2^; correspondingly, this difficult topic could further elucidate through this nationwide cancer registry database investigation. These results might raise questions about whether stroke prevention might be beneficial in patients with HCC when patients have longer expected survival times.

Previous studies investigating the association between HCC and stroke were typically only part of a subgroup analysis. Zöller et. al examined a cohort of patients with various cancers to investigate the association between the risk of various types of stroke and cancer^12^. In their subgroup analysis, patients with HCC only showed a higher risk of hemorrhagic stroke in the first 6 months after being diagnosed with HCC than cancer-free individuals, but there was a similar risk of ischemic stroke in patients with HCC and cancer-free individuals. In their analysis, they only considered conventional stroke risk factors as covariates in their multivariable analysis, such as age, hypertension, or diabetes mellitus. However, they had less emphasis on risk factors specific for patients with HCC, such as cirrhosis of the liver^9^, chronic kidney disease, or other factors related to bleeding tendency. Likewise, the association between stroke risk and various stages of cancer has been mentioned with respect to other cancers^4^, but there have been no reports concerning patients with HCC. Thus, our study only included patients with HCC and analyzed the relationship between the risk of stroke and HCC, considering stroke risk factors specific for patients with HCC and various HCC stages.

An important question for patients with HCC is whether they have adequate preserved liver function, even after surgical resection^2^. Although most patients with HCC often have impaired liver function due to cirrhosis, the proportion of noncirrhotic patients with HCC is also increasing because of increased surveillance of HCC in carriers of viral hepatitis and the rising incidence of non-alcoholic fatty liver disease^2,21^. Patients without cirrhosis often had better liver function and 5-year survival rates^22^; thus, they are likely to benefit most from prevention of cancer-associated complications, including stroke. Management for stroke prevention, either by antiplatelet or anticoagulant therapy, might cause major bleeding events such as gastrointestinal bleeding or intracranial hemorrhage^23^. These complications might be controllable in patients with preserved liver function. Our study indicates that patients without cirrhosis had a higher risk of stroke compared with cancer-free individuals; therefore, use of antiplatelets or anticoagulants might confer additional benefits in these patients for stroke prevention, especially during the first year after being diagnosed with HCC. Further studies are warranted to guide clinicians on how to balance the benefits of stroke prevention and the risk of major bleeding events in patients with HCC, especially those with preserved liver function.

This study also revealed that patients with advanced stages of HCC had a higher risk of stroke compared with cancer-free individuals. This manifestation was similar to other cancers mentioned in previous studies^4^. The positive relationship between the cancer stage and the amount of procoagulant materials secreted by cancer cells have been reported^24^, perhaps indicating why patients with advanced cancer were associated with a higher risk of stroke. Aside from stroke, portal vein thrombosis is a common cancer-associated complication in patients with HCC. Currently, while routine prophylactic anticoagulants are not recommended for every patient with HCC, personalized prophylactic anticoagulant use for portal vein thrombosis is recommended^10^. Patients with advanced stage HCC might obtain additional stroke prevention benefits when using prophylactic anticoagulants for portal vein thrombosis. Further studies are needed to assess whether prophylactic anticoagulants are advantageous for either portal vein thrombosis or stroke in patients with advanced stage HCC.

The present study has some limitations. First, not all stroke-related factors were adjusted. The administrative database did not provide all conventional stroke factors, thus information regarding body mass index, a history of smoking or drinking, diet, and physical habits were missing; these characteristics might be confounding risk factors for stroke in patients with HCC. Second, the database did not provide detailed laboratory data; hence, it was difficult to estimate the function of liver and coagulation in patients with HCC. Although we tried to balance the difference between patients with HCC and cancer-free individuals by a diagnosis of cirrhosis of the liver, chronic kidney disease, and major gastrointestinal bleeding, the liver status and coagulation function might be potential cofounding factors for stroke.

In conclusion, patients with HCC have a higher 1-year risk of stroke than those without cancer. In the era of increasing survival rate of patients with HCC, strategies for stroke prevention must be actively considered in these patients, especially in those without cirrhosis and those with stage 3 or stage 4 cancer.

## Methods

### Study design and data sources

The Taiwan Cancer registry (TCR) and the Taiwan National Health Insurance Research Database (NHIRD) were utilized to conduct a real-world, nationwide, registry-based cohort study. The TCR comprises nearly 90% of all patients with cancer in Taiwan and contains detailed information related to cancer, such as the staging, treatment, and specific dates of each procedure^13,14^. To obtain healthcare information from patients with cancer, identical encrypted codes were linked with the NHIRD from all 23.6 million enrollees. This TCR-NHIRD composite database provides comprehensive healthcare information, including all hospitalizations, emergency services, outpatient visits, and detailed medication prescription data. Furthermore, patients without cancer in the NHIRD were included as a cancer-free cohort. Both the NHIRD and TCR are maintained by the Health and Welfare Data Science Center of the Ministry of Health and Welfare in Taiwan^15^. The diagnostic and procedure codes of interest were the International Classification of Diseases, Ninth Revision, Clinical Modification (ICD-9-CM), before 2015, and the International Classification of Diseases, Tenth Revision, Clinical Modification (ICD-10-CM), after 2016. Additionally, the diagnostic codes for cancer types were based on the ICD for Oncology, Third Edition site recode classification (ICD-Oncology-3) in the TCR.

### Study population

Patients with HCC who were diagnosed with primary HCC, with an ICD-Oncology-3 C22 code in the TCR, from January 1, 2011, to December 31, 2015 were included. In patients with multiple primary cancers, only patients who had a first cancer diagnosis of HCC were recruited. A cancer-free cohort was also incorporated into the study; thus, each patient with HCC was matched to 2 age- and sex-matched patients without cancer from the TCR-NHIRD composite database.

Patients younger than 20 years old, those without complete basic information, those who were unable to be age- and sex-matched, or those having primary outcomes before a cancer diagnosis were excluded. Moreover, patients who died within 180 days of the cancer diagnosis were also excluded because different medical care policies might exist in terminal cancer patients with a shorter life expectancy.

### Outcomes

The date of the cancer diagnosis was defined as the index date in the HCC group, and identical dates were set as the index dates in the matched cancer-free individuals. The primary outcome was a composite outcome of either ischemic or hemorrhagic stroke, defined as any inpatient diagnosis of stroke with brain imaging examination. Previous manuscripts have validated these diagnostic criteria to clarify these outcomes in the Taiwan NHIRD^16,17^. An ischemic stroke diagnosis was defined as an inpatient diagnosis of ICD-9-CM codes 433, 434, and 436 or ICD-10-CM codes I63 and I67.89; a hemorrhagic stroke diagnosis was defined as an inpatient diagnosis with ICD-9-CM codes 430 and 431 or ICD-10-CM I60 and I61. Individuals were followed from the index date until the occurrence of stroke, either ischemic or hemorrhagic, or death, or until 1 year after the index date. In addition to the risk of stroke overall, we also analyzed the ischemic and hemorrhagic stroke separately as individual outcomes. The risk of stroke in patients with HCC was examined compared with cancer-free cohort. To investigate whether the risk of stroke in patients with HCC varied by the presence of cirrhosis, different cancer stages, or having cancer treatment, subgroup analyses were conducted based on these factors.

### Covariates

All comorbidities listing in Table 1 were defined as a discharge diagnosis or a diagnosis that was confirmed at least twice in an outpatient department before the index date, based diagnostic codes and procedure code. Charlson comorbidity index was calculated depending on baseline comorbidities; they represent the complexity of comorbidities in each patient^18^. Medication use related to stroke prevention was evaluated after the index date. Medication use was defined as at least 30 days of exposure to each drug during the study period. In patients with HCC, the cancer stage depended on pathological staging unless they had not received further curative operations; in that case, clinical staging was used. Cancer treatment included surgical therapy, chemotherapy, and radiation therapy.

### Sensitivity analysis

Since only part of the population was selected for analysis, there was a possibility of bias when analyzing data with propensity score matching. In order to validate the findings, sensitivity analyses were conducted including all eligible patients without matching; multivariable Fine and Gray competing risk regression models were used to adjust all covariates listed in Table 1.

### Statistical analysis

A 1:1 propensity score matching was performed using age, sex, income, Charlson comorbidity index, and comorbidities as listed in Table 1. Propensity score matching utilized a nearest-neighbor matching algorithm without replacement and used a caliper width equal to 0.2 of a standard deviation of the propensity score logit value. Standardized difference was used to assess the difference in baseline characteristics between groups, and a value of less than 0.2 was considered negligible. Regarding higher mortality among patients with HCC, a cumulative incidence function with death as a competing event was employed. The Gray’s test was utilized to examine the differences between cumulative incidence curves. Multivariable Fine and Gray competing risk regression models were used to measure the hazard ratios and corresponding 95% confidence intervals (CIs) for stroke with death as a competing event^19^. When analyzing ischemic stroke as an individual outcome, the event of hemorrhagic stroke was also considered as a competing risk, and vice versa. Age, sex, income, Charlson comorbidity index, comorbidities, and medication use related to stroke prevention were included and adjusted in the multivariable regression models. Statistical significance was defined as a two-tailed probability value < 0.05. All statistical analyses were performed using SAS, version 9.4 (SAS Institute Inc., Cary, NC) and Stata, version 14.0 (Stata Corporation, College Station, TX).

### Ethical approval

Our study were in accordance with the 1964 Helsinki declaration and its later amendments or comparable ethical standards, and thus this study was approved by the Institutional Review Board of Hualien Tzu Chi Hospital (IRB-107-06C).

### Informed consent

The Institutional Review Board agreed that informed consent could be waived because these database are encrypted databases.

## Supplementary Information

Supplementary Tables.

## Data Availability

The TCR and Taiwan NHIRD are an encrypted database that is regulated and maintained by the Health and Welfare Data Science Center at the Ministry of Health and Welfare in Taiwan. Therefore, the data set cannot be available publicly. Researchers interested in analyzing this data set can provide a formal application to the Taiwan Ministry of Health and Welfare to request access (website: https://dep.mohw.gov.tw/DOS/cp-2516-3591-113.html).
