# Supplementary material for: High 1-year risk of stroke in patients with hepatocellular carcinoma: a nationwide registry-based cohort study
Source: Sci Rep. 2021 May 17;11:10444. doi: 10.1038/s41598-021-89867-0 (PMC8128901; doi:10.1038/s41598-021-89867-0)
Supplement: Supplementary file 1 — Supplementary Tables. [file 41598_2021_89867_MOESM1_ESM.pdf]

High 1-year Risk of Stroke in Patients with Hepatocellular Carcinoma: A Nationwide Registry-based Cohort Study

Jin-Yi Hsu[a,b,], MD; Peter Pin-Sung Liu[a], MS; An-Bang Liu[b,c],MD, PhD; Huei-Kai Huang[b,d,e,*], MD; Ching-Hui Loh[a,b,d,*],MD, PhD

[a]Center for Aging and Health, Hualien Tzu Chi Hospital, Buddhist Tzu Chi Medical Foundation, Hualien, Taiwan

[b]School of Medicine, Tzu Chi University, Hualien, Taiwan

[c]Department of Neurology, Hualien Tzu Chi Hospital, Buddhist Tzu Chi Medical Foundation, Hualien, Taiwan

[d]Department of Family Medicine, Hualien Tzu Chi Hospital, Buddhist Tzu Chi Medical Foundation, Hualien, Taiwan

[e]Department of Medical Research, Hualien Tzu Chi Hospital, Buddhist Tzu Chi Medical Foundation, Hualien, Taiwan

Table S1. Baseline characteristics in patients with hepatocellular carcinoma and the cancer-free cohort without propensity score matching

	HCC cohort N = 33,468	Cancer-free cohort N = 66,936	Standardized difference
Age*	62.95 (11.7)	62.95 (11.7)	n/a
Male	2,3183 (69.3)	46,366 (69.3)	n/a
Income (NTD)			
< 15,800	6,241 (18.7)	14,331 (21.4)	0.069
15,800–25,000	17,586 (52.6)	30,509 (45.6)	0.140
25,001–35,000	6,105 (18.2)	12,737 (19.0)	0.020
35,001–45,000	3,536 (10.6)	9,359 (14.0)	0.104
45,001 and above	6,241 (18.7)	1,4331 (21.4)	0.069
Charlson comorbidity index*	3.45 (2.7)	1.59 (2.0)	0.775
Comorbidities			
Hypertension	15,583 (46.6)	26,338 (39.4)	0.146
Diabetes mellitus	10,771 (32.2)	12,710 (19.0)	0.306
Dyslipidemia	6,787 (20.3)	16,253 (24.3)	0.096
Atrial fibrillation	587 (1.8)	935 (1.4)	0.028
Valvular heart disease	382 (1.1)	149 (0.2)	0.112
Congestive heart failure	1,306 (3.9)	1,862 (2.8)	0.062
Coronary artery disease	4,324 (12.9)	7,940 (11.9)	0.032
Peripheral arterial occlusion disease	596 (1.8)	952 (1.4)	0.029
Cirrhosis	20,166 (60.3)	6,004 (9.0)	1.280
Chronic kidney disease	2,081 (6.2)	1,869 (2.8)	0.166
Major gastrointestinal bleeding	4,225 (12.6)	218 (0.3)	0.516
Medication use†			
Antithrombotic therapy	2,959 (8.8)	7,797 (11.7)	0.093
Anticoagulant	329 (1.0)	613 (0.9)	0.006
Antiplatelet	2,680 (8.0)	7,340 (11.0)	0.101
Cancer stage			
1	13,215 (39.5)	n/a	
2	8,905 (26.6)	n/a	
3	5,183 (15.5)	n/a	
4	2,323 (6.9)	n/a	

Unknown	3,842 (11.5)	n/a
Cancer treatment‡		
Yes	26,364 (78.8)	n/a
Surgical	20,589 (61.5)	n/a
Chemotherapy	4,105 (12.3)	n/a
Radiation therapy	2,303 (6.9)	n/a
No	7,104 (21.2)	n/a

Data are expressed as n (%) unless otherwise indicated.

* Expressed as the mean (SD).

† Medication use means a drug prescription for more than 30 days during the observation period.

‡ Cancer treatment means patients had either surgical therapy, chemotherapy, or radiation therapy.

Abbreviations: NTD: New Taiwan dollar; HCC; hepatocellular carcinoma; SD: standard deviation; n/a: not applicable

Table S2. Subgroup analyses to assess risk of ischemic stroke in patients with hepatocellular carcinoma compared with the cancer-free individuals according to the presence of cirrhosis, cancer stages, and cancer treatment

	aHR*	95% CI	p value
Cirrhosis			
Yes	1.15	0.79 –1.69	0.465
No	1.47	1.19–1.81	0.004
Cancer stage			
1	0.85	0.62–1.18	0.328
2	0.98	0.66–1.43	0.899
3	1.63	1.05–2.52	0.030
4	4.21	2.44–7.27	< 0.001
Unknown	1.96	1.22–3.12	0.005
Cancer treatment†			
Yes	1.29	1.05–1.58	0.016
No	1.70	1.15–2.52	0.008

* The hazard ratios were calculated using multivariable Cox proportional hazards regression model with adjustments for income, CCI, comorbidities listed in Table 1, medication use. Cancer-free control was the reference group.

† Cancer treatment means patients had either surgical therapy, chemotherapy, or radiation therapy.

Abbreviations: aHR: adjusted hazard ratio; CI: confidence interval; CCI: Charlson comorbidity index

Table S3. Subgroup analyses to assess hemorrhagic risk of stroke in patients with hepatocellular carcinoma compared with the cancer-free individuals according to the presence of cirrhosis, cancer stages, and cancer treatment

	aHR*	95% CI	p value
Cirrhosis			
Yes	1.24	0.87–1.75	0.229
No	1.73	1.42–2.09	< 0.001
Cancer stage			
1	0.93	0.69–1.26	0.650
2	0.97	0.68–1.39	0.878
3	2.22	1.51–3.27	< 0.001
4	4.90	3.00–7.99	< 0.001
Unknown	2.07	1.33–3.22	0.002
Cancer treatment†			
Yes	1.49	1.23–1.80	< 0.001
No	1.98	1.38–2.84	< 0.001

* The hazard ratios were calculated using a multivariable Cox proportional hazards regression model with adjustments for income, CCI, comorbidities, and medication use listed in Table 1. Cancer-free control patients comprised the reference group.

† Cancer treatment means patients had either surgical therapy, chemotherapy, or radiation therapy.

Abbreviations: aHR: adjusted hazard ratio; CI: confidence interval.
